# Mesenchymal stem cells of Oravka chicken breed: promising path to biodiversity conservation

**DOI:** 10.1016/j.psj.2023.102807

**Published:** 2023-05-25

**Authors:** Andrea Svoradová, Jaromír Vašíček, Vladimír Zmrhal, Eva Venusová, Aleš Pavlík, Miroslav Bauer, Lucia Olexiková, Vladimír Langraf, Petr Sláma, Peter Chrenek

**Affiliations:** ⁎ Institute of Farm Animal Genetics and Reproduction, NPPC, Research Institute for Animal Production in Nitra, Nitra, Slovakia; † Institute of Biotechnology, Faculty of Biotechnology and Food Science, Slovak University of Agriculture in Nitra, Nitra, Slovakia; ‡ Laboratory of Animal Immunology and Biotechnology, Department of Animal Morphology, Physiology and Genetics, Faculty of AgriSciences, Mendel University in Brno, Brno, Czech Republic; § Laboratory of Animal Physiology, Department of Animal Morphology, Physiology and Genetics, Faculty of AgriSciences, Mendel University in Brno, Brno, Czech Republic; # Department of Botany and Genetics, Faculty of Natural Sciences, Constantine the Philosopher University in Nitra, Nitra, Slovakia; ǁ Department of Zoology and Anthropology, Faculty of Natural Sciences, Constantine the Philosopher University in Nitra, Nitra, Slovakia

**Keywords:** chicken, native breed, stem cell, flow cytometry, cryopreservation

## Abstract

Mesenchymal stem cells (**MSCs**) are multilineage cells able to differentiate into other cell types. MSCs derived from bone marrow or compact bones are the most accessible stem cells used in tissue engineering. Therefore, the aim of this study was to isolate, characterize and cryopreserve MSCs of endangered Oravka chicken breed. MSCs were obtained from compact bones of the femur and tibiotarsus. MSCs were spindle-shaped and were able to differentiate into osteo-, adipo-, and chondrocytes under the specific differentiation conditions. Furthermore, MSCs were positive for surface markers such as CD29, CD44, CD73, CD90, CD105, CD146 and negative for CD34CD45 by flow cytometry. Moreover, MSCs demonstrated high positivity of “stemness” markers aldehyde dehydrogenase, alkaline phosphatase as well as for intracellular markers vimentin, desmin, α-SMA. Subsequently, MSCs were cryopreserved using 10% dimethyl sulfoxide in liquid nitrogen. Based on the results from the viability, phenotype, and ultrastructure assessment we can concluded that the MSCs were not negatively affected by the cryopreservation. Finally, MSCs of endangered Oravka chicken breed were successfully stored in animal gene bank, thus making them a valuable genetic resource.

## INTRODUCTION

Over the past few decades, research into the genetic diversity of endangered domestic animal breeds has been underway. The decline of genetic variability and the limited number of commercial hybrids indicate that genetic diversity in poultry is one of the most threatened genetic resources (Weigend and Romanov, 2001, 2002; Muir et al., 2008). There are several reasons to support the preservation of genes in native chicken breeds. Therefore, it is necessary to preserve the original chickens as a genetic resource that will reveal their special and distinctive genetic value for future breeding purposes. They can provide a source of unique alleles and facilitate the enrichment of genes associated with health and quality traits (Gandini and Oldenbroek, 1999; Mendelsohn, 2003). Another rationale for studies in this area is the careful observation of the genetic population's status, since native chicken breeds in conservation breeding programs are often kept in small populations and are therefore more affected by loss of genetic variation and inbreeding depression. Local chicken flocks usually have no provenance data, suffer from fluctuating population sizes (constraints), and lack properly planned breeding programs (Tixier-Boichard, 2009). These unique gene combinations make up a specific genotype are at risk of extinction, even though, they may represent potentially beneficial traits (Blackburn, 2006).

Based on the facts mentioned above, it is necessary to preserve various cell types of endangered breeds as a gene reserve for future purposes. One of such cell types represent nonhematopoietic, mesenchymal stem cells (**MSCs**). MSCs are multipotent stromal cells found in bone marrow cell population. They may be isolated from the bone marrow and are capable to differentiate into mesenchymal tissues such as adipose, cartilage and bone (Ayala-Cuellar et al., 2019). Among these, MSCs have attracted attention due to potential source, a high proliferation rate and ethical standards (Golchin et al., 2018). MSCs are plastic adherent and positive for cluster of differentiation (**CD**) CD73, CD90, CD105 surface antigens and also characterized by the lack of proteins expression such as CD45, CD34 (Sacchetti et al., 2007; Teresa Conconi et al., 2011; Lin et al., 2013; Cruz and Rocco, 2020). In chickens, MSCs can be used as feeder layer for primordial germ cells (**PGCs**) (Li et al., 2019), and thus support treatment for immunosuppressive disorder, such as infectious bursal disease virus (**IBDV**), which does not proliferate in chicken fibroblast cells (Majumdar et al., 1998). Moreover, they should be useful tool for examining underlying effects of vitamin D3, calcitriol (1,25-(OH)2D3) administration as well as a promising approach in immunomodulatory properties through the coculture in vitro with the pathogens (Gil et al., 2018). Therefore, it is desirable to cryopreserved MSCs for the purpose of subsequent use in possible studies of these disorders.

After all, the main goal of this study was to isolate, characterized and successful cryopreserved chicken MSCs of endangered Oravka breed into animal gene bank.

## MATERIALS AND METHODS

### Ethical Standards

Authors proclaim that all procedures conducted in this work abide by the ethical standards of the relevant national and institutional guidelines on the care and use of laboratory animals. The treatment of the animals was approved by the Ministry of Agriculture and Rural Development of the Slovak Republic no. SK U 18016* in accordance with the ethical guidelines presented in Slovak Animal Protection Regulation, RD 377/12, which conforms to European Union Regulation 2010/63. The experimental procedures were carried out strictly in accordance with the guidelines approved by the Animal Ethics Committee of Faculty of AgriSciences, Mendel University in Brno (Approval Number: 57199/2020-MZE-18134).

### Animals

For optimalization methods, clinically healthy chicken (*n* = 10) aged up to 21 d of the Ross 308 broiler chickens (Ross 308; Vykrm Trebic, Ltd., Chropyne, the Czech Republic) were obtained from a commercial hatchery and they were managed and cared for in accordance with guidelines broiler management handbook (Aviagen, 2014). However, Oravka chicken breed was used for cryopreservation into animal gene bank. Chickens were reared in a partially air-conditioned hall (14 h of light at 10 lux and 10 h of dark) at a breeding facility (NPPC, Research Institute for Animal Production Nitra, Lužianky, Slovak Republic), they were fed with commercial diet (KV; TEKRO Nitra, s.r.o., Slovakia) and the water was provided ad libitum using water feeders.

### Collection and Processing of the Biological Material

Chickens were humanely sacrificed at d 21. Femurs and tibiotarsus bones from both legs were obtained after cervical dislocation. Dissected legs were treated with 70% ethanol for few seconds and then kept in phosphate-buffered saline (**PBS**, Ca^−^ and Mg^−^ free; Biosera, Nuaille, France) containing 5% penicillin/streptomycin antibiotics (Thermo Fisher Scientific, Waltham, MA). Muscles and connective tissues around tibiotarsus and femurs were removed immediately using a scalpel and microdissecting scissors in a biosafety cabinet. Bone marrow (**BM**) inside the bone was flushed 3 times with PBS with a syringe to remove the bone marrow and hematopoietic cells. Afterward, the bones were chopped to smaller fragments of about 3 mm^3^. Bone fragments were washed 2 times in PBS and then put into the 5 mL Dulbecco's modified Eagle's medium (**DMEM**, Gibco, Thermo Fisher Scientific, Waltham, MA) supplemented with 20% fetal bovine serum (Sigma Aldrich, Gillingham, UK) and 1% penicillin/streptomycin antibiotics. Bone fragments were incubated at 37°C in a humidified incubator containing 5% CO_2_ in the atmosphere for 7 d. After 7 d rounded cells were visible. The culture medium was changed every 2 to 3 d. MSCs reached 90% confluency in about 6 to 7 d after their visibility. Then the cells were washed twice with 5 mL 1× PBS, dissociated with 0.05% Trypsin-EDTA (Mediatech Inc., Richmond, VA) for 3 min, counted using EVE Automatic cell counter (NanoEntek, Seoul, South Korea) and subcultured at a ratio of 16,000 cells/cm^2^ on 25 cm^2^ culture flasks. Cells were cultured until passage 3 (**P3**). Cell aliquots from P3 were used for the phenotyping of cells using flow cytometry. Moreover, during the culture cell morphology was observed using Zeiss Axio Observer.Z1/7 microscope (Carl Zeiss Slovakia, Bratislava, Slovakia).

### Viability Assessment of MSCs

The flow cytometry method for viability analysis was used. After trypsinization, the cells were divided into 2 tubes and washed in PBS^+^ (with Ca; Mg, Gibco BRL, Billings, MO). The samples were centrifuged for 5 min at 300 × *g*. After centrifugation, the cell pellet to be stained was resuspended in 98 µL of Annexin V (**AnV**, Roche) Binding buffer and 2 µL of AnV dye was added. The tubes were incubated for 15 min in the dark at room temperature. We added 1 mL of Binding buffer to the tubes and centrifuged at 300 × *g* for 5 min. The pellets were resuspended in 200 µL of Binding buffer. Prior to analysis, we added 5 µL of 7-AAD (eBioscience) and incubated for 10 min in the dark at room temperature. We expressed the results by the percentage of living cells (AnV^−^/7-AAD^−^), apoptotic cells (AnV^+^/7-AAD^−^), and dead cells (AnV^−^/7-AAD^+^; AnV^+^/7-AAD^+^). Samples were evaluated with a FACS Calibur flow cytometer (BD Biosciences, San Jose, CA) and Cell Quest Pro software (BD Biosciences). In each sample, 25,000 events (cells) of MSCs in P3 were analyzed.

### Detection of Surface and Intracellular Markers Using Flow Cytometry

To confirm the origin of chicken MSCs, the detection of the cell surface and intracellular markers was performed by an antibody based immunofluorescent staining. After centrifugation, the cell (P3) pellet was resuspended, and the cell suspension was aliquoted approximately 10^6^ cells per tube. Next, the cells were resuspended in inactivated chicken serum (Gibco BRL) to block Fc receptors. Prefixation and permeabilization with acetone:methanol (1:1) mixture was applied for intracellular cytoplasmic markers (vimentin, desmin, α-SMA). The cells were double-stained using bellow listed primary antibodies (Table 1) and goat anti-mouse IgG-FITC secondary antibody (Bio-Rad, Hercules, CA). The labeled samples were incubated for 15 min on ice in the dark and after incubation, the samples were washed with PBS and centrifugated (5 min, 300 × *g*). To exclude the dead cells from the analysis, samples were costained with dead cell marker 7-AAD. Cells were analyzed using a FACS Calibur device (BD Biosciences, San Jose, CA) and Cell Quest Pro software (BD Biosciences). At least 25,000 events were analyzed for each sample. Unstained samples were used as control samples to gate the positive cells according to the increased fluorescent intensity (Figure 3).

**Table 1:** List of primary antibodies used for flow cytometry and confocal microscopy.

Marker	Host/isotype	Clone	Conjugate	Company
CD73	Mouse IgG1	AD2	FITC	BD Biosciences
CD90	Mouse IgG1	5E10	FITC	BD Biosciences
CD105	Rabbit IgG	CF647	APC	BiorByt
CD29	Mouse IgG1	P4G11	FITC	Merck
CD44	Mouse IgG1κ	AV6	APC	SouthernBiotech
CD45	Mouse (BALB/c) IgMκ	LT40	PE	SouthernBiotech
CD34	Rabbit IgG	Polyclonal	PE	Bioss
CD146	Mouse IgG2b Kappa	c264	FITC	Novus Biologicals
Vimentin	Mouse IgG2a	Vim 3B4	Purified	Dako Cytomation
Desmin	Mouse IgG1	D33	Purified	Dako Cytomation
α-SMA	Mouse IgG2a	1A4	Purified	Dako Cytomation
Marker	Host/Isotype	Clone	Conjugate	Company
CD73	Mouse IgG1	AD2	FITC	BD Biosciences
CD90	Mouse IgG1	5E10	FITC	BD Biosciences
CD105	Mouse IgG1	266	FITC	BD Biosciences
CD29	Mouse IgG1	P4G11	FITC	Merck
CD44	Mouse IgG1	W4/86	Purified	Bio-Rad
CD45	Mouse (BALB/c) IgMκ	LT40	PE	SouthernBiotech
CD34	Rabbit IgG	Polyclonal	PE	Bioss
CD146	Mouse IgG2b Kappa	c264	FITC	Novus Biologicals

α-SMA, α smooth muscle actin. Cells stained with the purified antibodies were subsequently incubated with proper secondary antibodies. Cells stained with the purified antibodies were subsequently incubated with proper secondary antibodies.

Moreover, aldehyde dehydrogenase (**ALDH**) activity as a marker of “stemness” was assessed using the ALDEFLUOR kit (STEMCELL Technologies, Vancouver, BC, Canada) and evaluated using flow cytometry. Briefly, cells (P3) were incubated with an Aldefluor substrate (15 min; 37°C) with or without the ALDH inhibitor diethylamino benzaldehyde (**DEAB**) in accordance with the manufacturer's guidelines. Stained cells were analyzed by a flow cytometer (FACSCalibur, BD Biosciences). At least 25,000 cells were analyzed in each sample.

### Detection of Surface and Intracellular Markers Using Confocal Microscopy

For the visualization of the selected MSCs markers an immunofluorescence assay was performed. Briefly, approximately 3 × 10^4^ cells from the passage 2 (**P2**) were resuspended in culture medium and allowed to adhere to a microscopic slide placed into a 4-well plate (NUNC) at 37°C in a 5% CO_2_ humidified atmosphere until reaching 80% confluency. For surface markers (CD73, CD90, CD105, CD146, CD44, CD45, CD34), the cells were prefixed using an IC Fixation Buffer (Thermo Fisher Scientific, Waltham, MA). Prefixation and permeabilization with acetone:methanol (1:1) mixture was applied for intracellular cytoplasmic markers (vimentin, desmin, α-SMA). Thereafter, the cells were gently washed with PBS and incubated with primary antibodies overnight. Afterward, cells were washed with PBS and incubated with an adequate secondary antibody as described above. Following the final cell wash with PBS, 4 µL of Vectashield antifade mounting medium containing DAPI nuclear stain (Vector Laboratories, Burlingame, CA) were pipetted on a microscope slide. Lastly, a coverslip with adhered cells was carefully placed on a microscope slide with the cell-coated side down. Stained cells were evaluated using an LSM 700 laser scanning confocal microscope (Carl Zeiss Slovakia, Bratislava, Slovak Republic). Due to better fluorescent signal FITC conjugates were used for confocal microscopy. Intracellular markers were identically as previous.

### RT-PCR

RT-PCR analyses were carried out to detect mRNA expression of specific cell markers. Total RNA from 3 to 5 × 10^6^ chicken stem cells as well as from chicken bone marrow (positive control) was isolated using TRI Reagent RT (Molecular Research Center, Cincinnati, OH) according to the manufacturer's protocol. The purity of extracted RNA was determined by UV spectrophotometry at 260/280 nm ratio and the integrity of RNA was checked by electrophoresis in 1% agarose gel. To destroy contaminating DNA, before reverse transcription, RNA samples were treated with the dsDNase (Thermo Fisher Scientific). The first-strand cDNA was synthesized using Maxima H Minus First Strand cDNA Synthesis Kit (Thermo Fisher Scientific) with 1.5 µg of total RNA from each sample and oligo (dT)_18_ in a total volume of 20 µL. The reaction was performed at 50°C for 30 min, and terminated at 85°C for 5 min. A PCR was performed in 20 µL reactions containing 1 µL cDNA, 4 µL of 5× MyTaq reaction buffer, 1 U of MyTaq HS DNA polymerase (Bioline, Memphis, TN), and 5 pmol of each primer for tested markers (Table 2) using C1000 Thermal Cycler (Bio-Rad). Chicken β-2-microglobulin (**B2M**) was applied as a reference gene, and the amplification protocol for all genes was as follows: an initial denaturation and activation of Taq DNA polymerase at 95°C for 2 min, followed by 35 cycles of denaturation at 95°C for 15 s, annealing at 60°C (58°C in the case of CD45) for 15 s and polymerization at 72°C for 15 s (30 s in the case of CD45). The final polymerization step was extended to 5 min at 72°C. PCR products were electrophoretically separated in 2% agarose gel in TAE buffer. PCR product sizes and primer sequences used in this study are listed in Table 2. Primers for CD29, CD44, CD73, CD90, CD105, CD146, B2M, alkaline phosphatase (**ALPL**) and ALDH were designed using the Primer-BLAST at the NCBI website (Ye et al., 2012).

**Table 2:** PCR product sizes and primer sequences.

Gene	Size (bp)	Forward primer	Reverse primer	Reference
B2M	188	5′ ACCCACCCAAGATCTCCATC 3′	5′ GTAGACCTGCGGCTCCTTC 3′	NM_001001750.4^1^
ALPL	196	5′ AACGGCCCTGGCTATAAGAT 3′	5′ GGGGGATGTAGTTCTGCTCA 3′	XP_046759065.1^1^
ALDH	153	5′ TCTTTAACCCCGCAAATGAG 3′	5′ TGTTCAAGAGCCTTCCTCGT 3′	XP_040528474.1^1^
CD73	149	5′ CCCATATCCCTTCATGGTTG 3′	5′ CCAGCAGGATAGGATTTCCA 3′	XM_040669143.2^1^
CD90	160	5′ AAAGCACCATCAGCGTCTCT 3′	5′ ATCTGGTTGCCGGTGTAGTC 3′	XM_046932252.1^1^
CD105	126	5′ GAGCTGAAGGACCCACAGAG 3′	5′ CTCACGGAAGAGGACCTCAG 3′	NP_001074356.2^1^
CD44	133	5′ TAACGTCACAACCAGGGACA 3′	5′ AGCTTTTTCTTCTGCCCACA 3′	XM_040700645.2^1^
CD29	166	5′ AATGTGGTGCATGCAGATGT 3′	5′ TTCTTGCATACGCACTGTCC 3′	XM_046917243.1^1^
CD146	115	5′ ACAGCTGGCAGGATATGACC 3′	5′ TCGTCCAAGTCCAGTGTCTG 3′	NP_001382961.1^1^
CD34	239	5′ GTGCCACAACATCAAAGACG 3′	5′ GGAGCACATCCGTAGCAGGA 3′	Adhikari et al., 2019
CD45	574	5′ CACTGGGAATTCGAGAGAAA 3′	5′ CTGGTCTGGATGGCACTTT 3′	Khatri et al., 2009

1 NCBI reference sequence; ALDH, aldehyde dehydrogenase; ALPL, alkaline phosphatase; B2M, β-2 microglobulin.

### Differentiation

To evaluate the multipotent character of chicken MSCs, cells were differentiated into 3 basic line ages (adipogenic, chondrogenic, and osteogenic) using standard induction media. Differentiation into adipogenic, chondrogenic and osteogenic lineages was performed in accordance with the manufacturer's instructions of commercially available kits (StemPro Adipogenesis, StemPro Chondrogenesis, StemPro Osteogenesis; Thermo Fisher Scientific). Briefly, MSCs were cultured till P2 as described above, subsequently cells were detached and re-seeded into 6 cm^2^ tissue culture plates with a density of 1.0 × 10^4^ cells per cm^2^. After 48 h, cells became subconfluent (about 80%), culture medium was discarded, cells were washed with PBS and the medium was replaced with a mesenchymal stem cell osteogenic, chondrogenic and adipogenic differentiation medium (PromoCell, Heidelberg, Germany). Differentiation of chicken stem cells into above mentioned lineages was performed according to the manufacturer's instructions under standard growth condition (37°C; 5% CO_2_). The medium was changed after every 3 d. After 14 d of induction (adipogenic, chondrogenic) and 21 d (osteogenic) induction, cells were histologically stained with Oil-Red-O, Alizarin-Red and Safranin-O to evaluate the differential potential, as described in our previous studies (Kulíková et al., 2019; Vašíček et al., 2020; Tomková et al., 2021).

### Transmission Electron Microscopy

For a more comprehensive overview of cell ultrastructure of MSCs, the analysis using transmission electron microscopy (**TEM**) was conducted. The cells (P3) were immediately fixed in Karnovsky fixative solution (2% paraformaldehyde and 2.5% glutaraldehyde in 0.15 mol/L sodium cacodylate buffer, pH 7.1–7.3) during 1 h at 4°C and then washed 3 times in a cacodylate buffer for 15 min. Pellets were embedded into 2% agar and postfixed in 1% osmium tetraoxide in a cacodylate buffer during 1 h. Samples were then dehydrated in 50, 70, 95, and 100% of acetone for 2, 10, 30, and 60 min, respectively, and embedded in Poly/Bed resin (Polysciences Inc., Warrington, PA). The blocks of MSCs were cut into semithin sections (1 μm) stained with a methylene blue, and ultrathin sections (70 nm) were placed on nickel grids, contrasted with uranyl acetate and lead citrate, and examined under a transmission electron microscope (JEM2100, JEOL, Japan) operating at 200 kV. For each group, electromicrographs were recorded at a microscope magnification of 1,900×.

### Cryopreservation

Chicken MSCs were frozen in freezing solution composed of medium used for culture and 10 % DMSO (D2650, Sigma-Aldrich). One million of cells were mixed with 1.5 mL of freezing solution and placed into cryovials. Cryovials were frozen at slow rate by reducing the temperature by 1°C per min in Mr. Frosty container (Thermo Scientific Nalgene, Rochester, NY) and stored for 24 h at −80°C. After 24 h the cryovials were immersed into liquid nitrogen and stored for 1 mo. MSCs were thawed in water bath at 37°C for 30 to 60 s.

### Statistical Analysis

For normality distribution Shapiro-Wilk *W* test was used. The experiment was repeated 10 times and data were evaluated using GraphPad Prism version 9.2.0 for Windows (GraphPad Software, San Diego, CA) with 1-way ANOVA followed by Sidak's test for multiple comparisons. Results are expressed as the mean ± SD. *P* values at *P* < 0.05 were considered as statistically significant.

## RESULTS

### Morphology and Viability of MSCs

**Figure 1:**
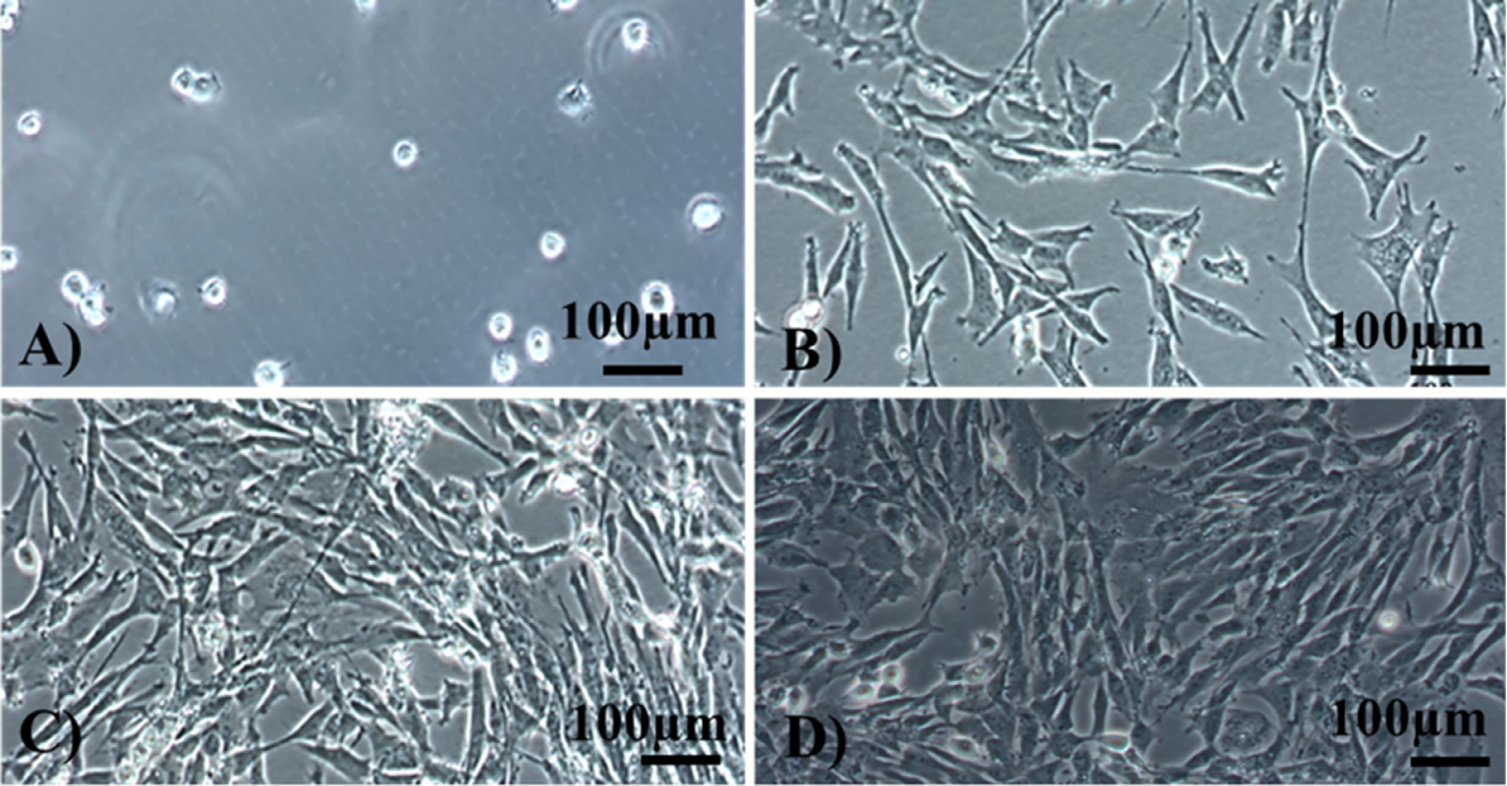
Morphological changes in MSCs during the culture. (A) Cells started to adhere to culture flasks after seeding; (B) observation of small colonies after 72 h in culture; (C) the cells reached confluency approximately 50 to 60% on the fifth day; (D) a homogenous monolayer of MSCs after eighth day. Magnification 200×, phase contrast (scale bar = 100 µm).

MSCs were isolated from 21-day-old broiler compact bones of the tibia and femur. After the 7 d of bone fragments culture, the cells with round shape were observed (Figure 1A). After additional 24 h of culture cells began to cluster into small colonies and started to adhere to tissue culture flasks, while their morphology changed into spindle-shaped (Figure 1B). The medium was replaced every 2 to 3 d to remove nonadherent cells. Approximately on the third day after cell attachment, cells began to rapidly proliferate and reached about 60 to 70% confluency (Figure 1C). After next 6 to 7 d of culture, the cells reached 90% confluency and the culture consisted of a homogenous monolayer of fibroblast-like cells (Figure 1D) was observed. Then, the cell monolayer was detached, and cells were re-seeded as subsequent passage into the new flasks.

**Figure 2:**
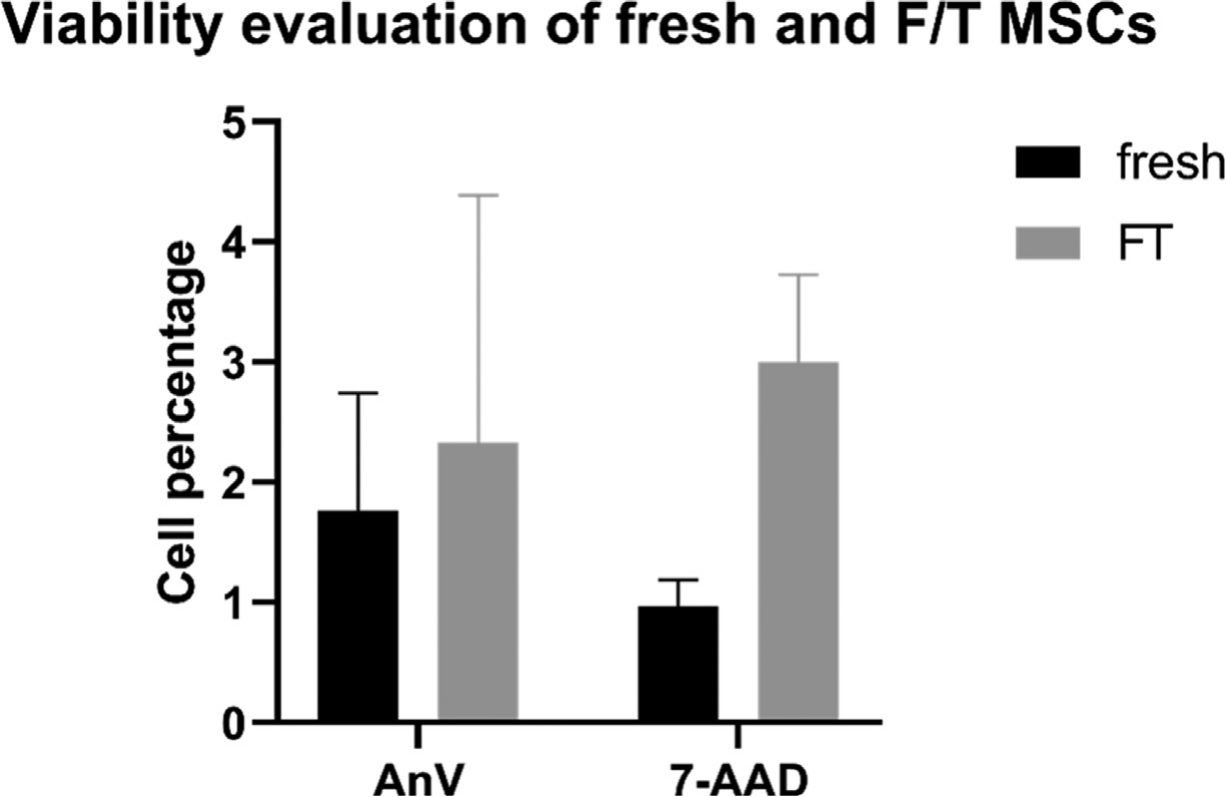
Proportion of apoptotic and dead MSCs in fresh (P3) and frozen/thawed samples. F/T, frozen/thawed.

The viability of MSCs (P0–P3) remained sufficient during the culture as the proportion of live cells varied from 90 to 97% (data do not show). The proportion of apoptotic and dead cells in the P3 passage was negligible (less than 2 or 1%, respectively, Figure 2). Similarly, proportion of apoptotic and dead cells in frozen/thawed MSCs samples did not exceed 5% (Figure 2). There were no significant differences between groups.

### Detection of the Expression of Surface and Intracellular Markers Using Flow Cytometry

The phenotypic analysis of MSCs (P3) showed high positivity of CD73, CD90, CD105 CD29, CD44, CD146 as well as intracellular markers - vimentin, desmin and α-smooth muscle actin (**α-SMA**). The activity of ALDH was also detected at high level (more than 70%). Markers of hematopoietic lineage (CD34 and CD45), used as a negative control, were not detected (Table 3).

**Table 3:** Detection of the expression of markers using flow cytometry.

Marker expression	
CD73	95.56 ±3.61
CD90	96.70 ±2.58
CD105	91.49 ±7.85
CD29	90.07 ±5.38
CD44	91.16 ±4.49
CD45	ND
CD34	ND
CD146	86.09 ±6.75
Vimentin	86.20 ±2.06
Desmin	61.25 ±1.25
α-SMA	84.87 ±1.85
ALDH	74.87 ±1.61

α-SMA, α smooth muscle actin; ALDH, aldehyde dehydrogenase. The marker expression is presented as the mean (%) ± SD. ND, marker expression was not detected.

### Detection of Surface and Intracellular Markers Using Confocal Microscopy

The confocal microscopy confirmed the MSCs phenotype and the expression of all surfaces (Figure 4) and intracellular markers (vimentin, desmin, and α-SMA; Figure 5) detected using flow cytometry.

### RT-PCR

The expression of surface markers was assessed at the mRNA level using the RT-PCR method. The following cell surface markers were examined: CD29, CD44, CD73, CD90, CD105, CD146, CD34, and CD45. Expression of other stem cell-specific markers (ALDH, ALPL) were also monitored. The B2M was used as a reference gene. The results of RT-PCR analyses confirm that chicken MSCs express all CD surface markers characteristic for MSCs (CD29, CD44, CD73, CD90, CD105, and CD146). The markers of the hematopoietic line (CD34 and CD45) were also tested in MSCs sample (Figures 6 and 7), whereas MSCs weakly expressed CD34 but not CD45, however CD45 was detected in chicken bone marrow used as a positive control.

### MSCs Differentiation

We evaluated the differentiation potential of the cells based on the positive histological staining of fresh cells in P3. Stained lipid droplets in the cytoplasm using Oil-Red-O confirmed adipogenic differentiation (Figure 8B). Positive staining of proteoglycan deposits with Safranin-O confirmed chondrogenesis (Figure 8D). Successful osteogenesis was verified by the detection of calcium aggregates (Figure 8F) stained with Alizarin-Red-S. As a control, noninduced cells were exposed to the staining with the appropriate dyes in recommended time intervals (Figure 8A, C, and E).

### Transmission Electron Microscopy

In order to control the ultrastructure, transmission electron microscopy analysis was performed on fresh and frozen/thawed MSCs (P3). The cells showed a round shape, their cytoplasmic membrane on the surface ran into numerous protrusions. Large eccentrically localized (mostly euchromatic) oval or lobed-shaped nuclei with numerous invaginations were well observable in the cells. We confirmed the presence of active large nuclei of the reticular type with a well visible fibrillar and granular component. Numerous dense oval mitochondria with crests, and vacuoles were found in the cytoplasm. In the case of frozen/thawed MSCs we observed damaged MSCs cells (triangle). However, only few cells were damaged after cryopreservation (Figure 9).

## DISCUSSION

MSCs are important source for therapy in cell regenerative medicine specially for repairing damaged tissues in various diseases, both in animal models as well as in human (Nakamura et al., 2013; Kawai et al., 2015; Oh et al., 2018; Selvasandran et al., 2018; Xu et al., 2018; Kim et al., 2019).

As far we know, this is the first study reported cryopreservation of MSCs derived from compact bones of endangered Oravka chicken breed. Here, we presented methodology for the isolation, cultivation, phenotype characterization, differentiation and cryopreservation of chicken MSCs. Commonly, from a morphological point of view, these cells show a fibroblast-like shape, which changes during the culture from round to spindle-shaped in many animal models and sources (Gayathri et al., 2016; Arrizabalaga and Nollert, 2017; Palumbo et al., 2018; Zomer et al., 2018; Bourebaba et al., 2019; Elashry et al., 2019; Machado et al., 2019; Zhan et al., 2019). MSCs well proliferate are positive for specific surface and intracellular markers and display osteogenic, adipogenic, and chondrogenic differentiation as previously described in Dominici et al. (2006).

In the present study, MSCs isolated from compact bones showed logarithmic phase and a plateau phase in about 7 to 8 d, as well as spindle-shaped morphology (Figure 1) similarly recorded by Khatri et al. (2009), Bai et al. (2013), Adhikari et al. (2019). The phenotype of chicken MSCs is thoroughly characterized in various studies (Khatri et al., 2009; Bai et al., 2013; Adhikari et al., 2019). Due to weak availability of stem cell-specific markers in poultry, researchers have to rely on reports of cell surface markers in mammalian species. Verification of cell origin is an important quality control step to eliminate a contamination of MSCs with other cell types.

In general, these cells are defined as CD29^+^CD44^+^CD73^+^CD90^+^CD105^+^CD34^−^CD45^−^ (Adhikari et al., 2019). In present study, a similar phenotype, even though with small differences, was confirmed also for chicken MSCs by flow cytometry and PCR methods on the mRNA level. The positive expression of surface and intracellular markers and the antibodies specificity were also verified by confocal microscopy. Since a limited number of markers were analyzed in the chicken studies, additional phenotype of chicken MSCs was required. The expression of intracellular markers vimentin, desmin, αSMA, and the activity of ALPL and ALDH was also recorded. The level of ALPL and ALDH is widely used as a marker of the cells “stemness” due to its relation to self-renewal and differentiation capabilities (Vassalli, 2019). According to the above-described data and previously published results, there were some differences between these methods in the expression of one marker. Some discrepancies in CD34 expression analyzed using PCR method were recorded. While CD34 marker was not express in previous studies (Khatri et al., 2009; Bai et al., 2013; Adhikari et al., 2019) related to chicken MSCs, in our study PCR method confirmed MSCs positivity to CD34. However, other proposed primers were used to detect CD34 expression. On the other hand, flow cytometry and confocal microscopy did not affirm the positivity for CD34 in MSCs.

However, some authors considered CD34 as a marker of other nonhematopoietic cell linages such as MSCs, interstitial dendritic cells, and epithelial progenitors (Sidney et al., 2014). On the other hand, while CD34 expression was positive in MSCs, another marker of hematopoietic lineages CD45 did not express in MSCs. Previous publications regarding chicken MSCs have not analyzed intracellular (vimentin, desmin, actin) markers (Khatri et al., 2009; Bai et al., 2013; Adhikari et al., 2019). Therefore, the expression of these markers in chicken MSCs was also recorded in our study. MSCs showed high positivity of these markers detected by flow cytometry and confocal microscopy.

Commonly, MSCs demonstrate multilineage differentiation potential into 3 lineages: chondrocytes, osteocytes and adipocytes under the standard culture conditions in specific differentiation media. Commercial kits offer different types of media with specific supplements to achieve cell differentiation (Almaki and Agrawal, 2016). In this work, we differentiated MSCs into 3 lineages using commercially available differentiation kits. Histological staining showed the successful differentiation of cells into osteogenic (Alizarin Red), adipogenic (Oil red) and chondrogenic lines (Safranin-O) (Figure 8) as in previous studies (Khatri et al., 2009; Bai et al., 2013; Adhikari et al., 2019).

In order to maintain the highest quality of the cryopreserved chicken MSCs, an optimal freezing protocol has to be used. Here, as a cryoprotectant, 10% DMSO was used, and the temperature was decreasing gradually 1°/min^−1^ in the controlled device. The thawed chicken cells maintained high viability and low apoptosis rate (Figure 2) even after 1 mo of storage in liquid nitrogen. Similarly, other authors used 10% DMSO as a part of freezing media (Bárcia et al., 2017; Somal et al., 2017; Lohan et al., 2018; Khan et al., 2019; Rogulska et al., 2019; Salmenkari et al., 2019; Bharti et al., 2020; Yea et al., 2020) in mammalian model.

In summary, in the case of mammals, information is known about the phenotype and functionality of the cells. However, there are few studies focusing on avian cells. Therefore, in the presented work, we focused on mesenchymal cells of poultry. From our results, we found that MSCs can be isolated from compact bones by culturing them for 8 d. In addition, MSCs express intracellular markers vimentin, desmin, actin, ALDH, and ALPL, which expanded the field of their deeper knowledge and usability. Morphology, phenotype, differentiation potential as well as ultrastructure make them a suitable model for various fields of research. Since MSCs have immunomodulatory effect, regulate hematopoiesis, are capable of multilineage differentiation to osteogenic lineages, etc., are suitable for better understanding the process of the infectious bursal disease virus as well as mineralization during osteogenesis.

## CONCLUSIONS

In conclusion, we can sum that we successfully isolated, cultured, characterized, and cryopreserved MSCs of endangered Oravka chicken breed. The viability and phenotype of chicken MSCs were not affected by the cryopreservation, thus making them a valuable source for animal gene banks.

**Figure 3:**
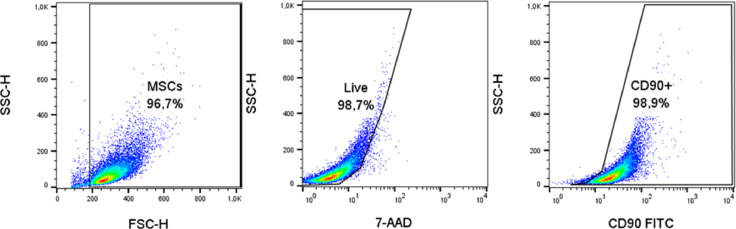
Illustrative strategy of flow-cytometric analysis.

**Figure 4:**
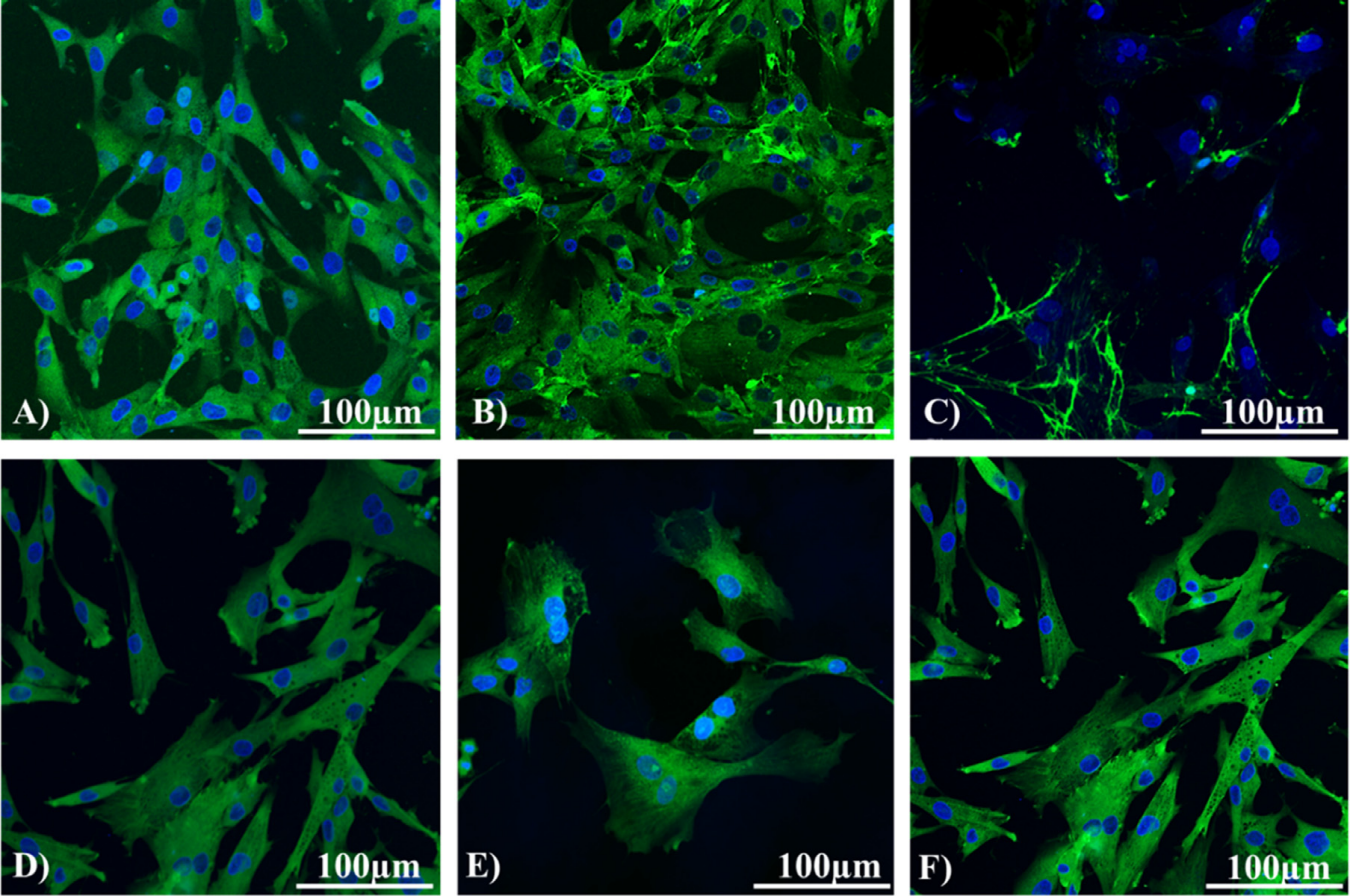
Immunofluorescence of selected surface markers of chicken MSCs (scale bar = 100 µm). (A) CD73, (B) CD90, (C) CD105, (D) CD44, (E) CD146, (F)CD29.

**Figure 5:**
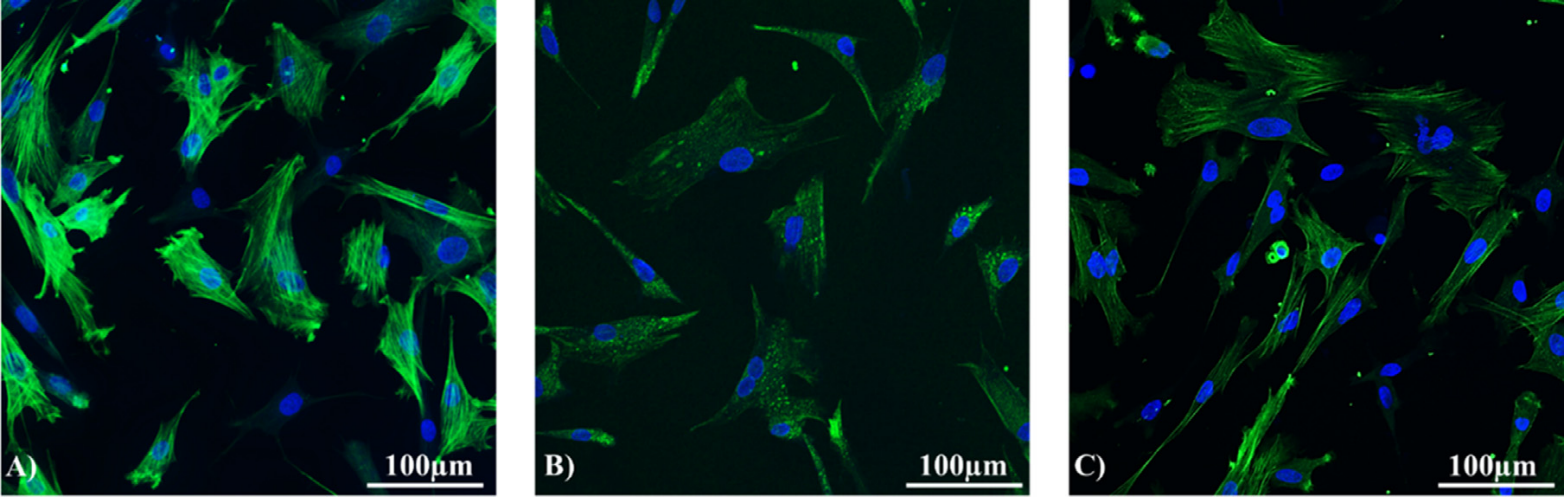
Immunofluorescence of selected intracellular markers of chicken MCSs (scale bar = 100 µm). (A) Vimentin, (B) Desmin, (C) Actin.

**Figure 6:**
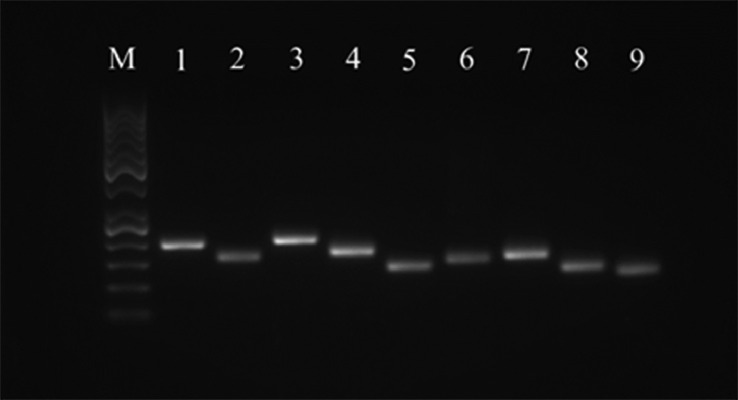
RT-PCR analysis of surface and stem cell-specific markers gene expression in chicken MSCs in P3. Line M—50 bp DNA ladder (Thermo Fisher Scientific); line 1—β-2 microglobulin (B2M); line 2—ALDH; line 3—ALPL; line 4—CD29; line 5—CD44; line 6—CD73; line 7—CD90; line 8—CD105; line 9—CD146.

**Figure 7:**
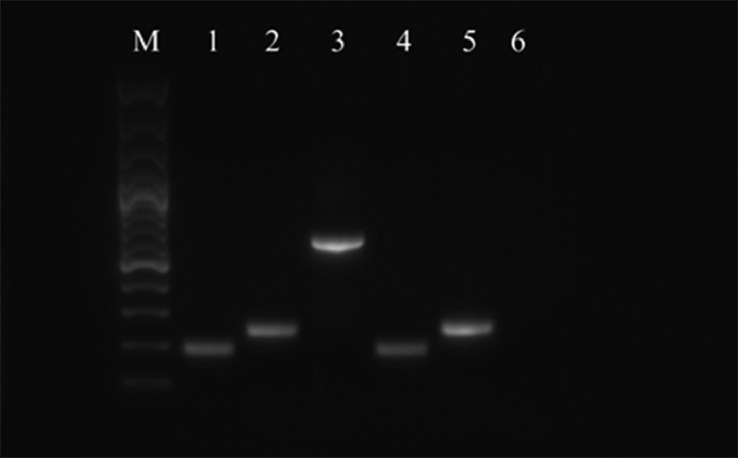
RT-PCR analysis of surface markers gene expression in chicken MSCs (P3) and bone marrow (BM) as a positive control. Line M—100 bp DNA ladder (Thermo Fisher Scientific); line 1—β-2 microglobulin (B2M) in BM; line 2—CD34 in BM; line 3—CD45 in BM; line 4—B2M in MSCs; line 5—CD34 in MSCs; line 6—CD45 in MSCs.

**Figure 8:**
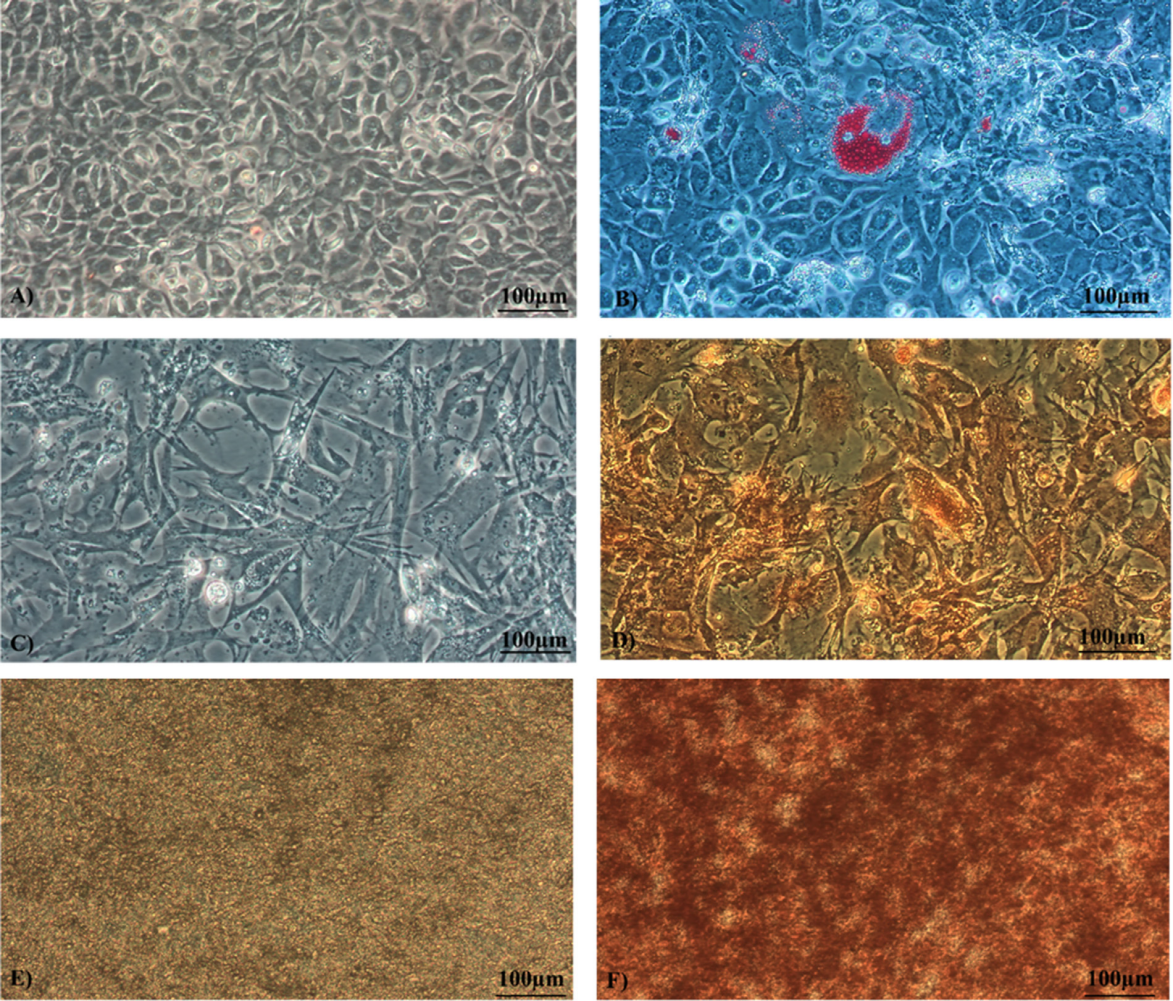
Histological staining of MSCs. (A, C, E) Nondifferentiated cells remained unstained; (B) lipid drops are stained red by Oil-Red-O; (D) deposits of proteoglycan in the chondrogenic-induced sample are stained with Safranin-O; (F) red dye Alizarin-Red-S identifies accumulation of calcium aggregates (scale bar = 100 µm).

**Figure 9:**
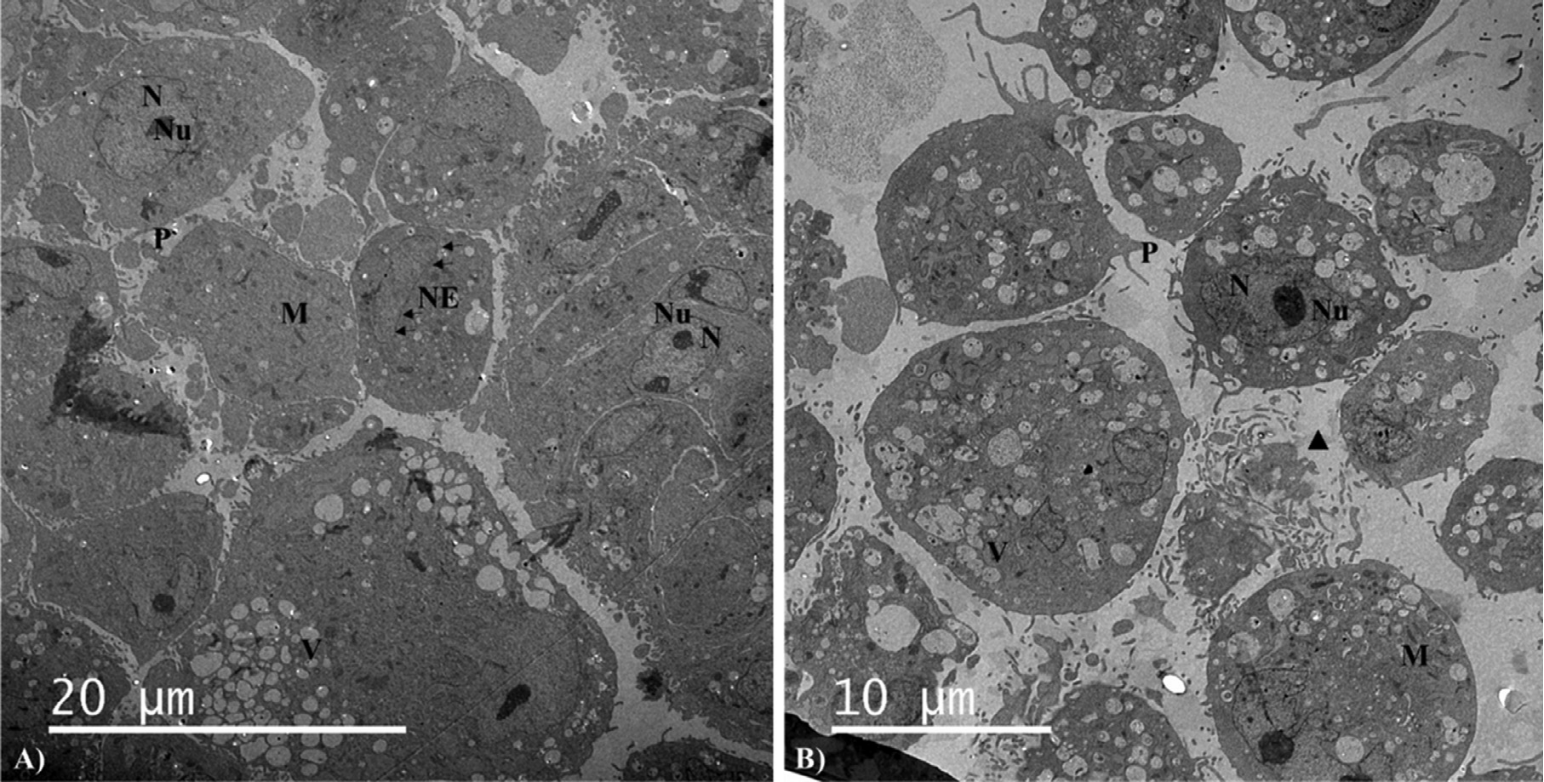
Ultrastructure of fresh (A) MSCs and frozen/thawed (B) MSCs. P, pseudopodia; NE, nuclear membrane; Nu, nucleolus; N, nucleus; V, vacuole; M, mitochondria (magnification 1,900×; scale bar = 10 µm; 20 µm).
